# Conundrum for Psoriasis and Thyroid Involvement

**DOI:** 10.3390/ijms24054894

**Published:** 2023-03-03

**Authors:** Cristina-Ilinca Cira, Mara Carsote, Claudiu Nistor, Aida Petca, Razvan-Cosmin Petca, Florica Sandru

**Affiliations:** 1 Department of Dermatovenerology, “Carol Davila University” of Medicine and Pharmacy, “Elias” University Emergency Hospital, 011461 Bucharest, Romania; 2 Department of Endocrinology, “Carol Davila” University of Medicine and Pharmacy, “C.I. Parhon” National Institute of Endocrinology, 011461 Bucharest, Romania; 3 Department 4—Cardio-Thoracic Pathology, Thoracic Surgery II Discipline, “Carol Davila” University of Medicine and Pharmacy, Thoracic Surgery Department, “Dr. Carol Davila” Central Emergency University Military Hospital, 011461 Bucharest, Romania; 4 Department of Obstetrics and Gynecology, “Carol Davila” University of Medicine and Pharmacy, “Elias” University Emergency Hospital, 011461 Bucharest, Romania; 5 Department of Urology, “Carol Davila” University of Medicine and Pharmacy, “Prof. Dr. Theodor Burghele” Clinical Hospital, 050659 Bucharest, Romania

**Keywords:** psoriasis, thyroid, thyroiditis, autoimmune, antibodies, Hashimoto’s thyroiditis, Basedow disease, thyroid cancer, pathogenic

## Abstract

Strategies concerning thyroid anomalies in patients confirmed with psoriasis, either on clinical level or molecular levels, and their genetic findings remain an open issue. Identification of the exact subgroup of individuals that are candidates to endocrine assessments is also controversial. Our purpose in this work was to overview clinical and pathogenic data concerning psoriasis and thyroid comorbidities from a dual perspective (dermatologic and endocrine). This was a narrative review of English literature between January 2016 and January 2023. We included clinically relevant, original articles with different levels of statistical evidence published on PubMed. We followed four clusters of conditions: thyroid dysfunction, autoimmunity, thyroid cancer, and subacute thyroiditis. A new piece of information in this field was the fact that psoriasis and autoimmune thyroid diseases (ATD) have been shown to be related to the immune-based side effects of modern anticancer drugs—namely, immune checkpoint inhibitors (ICP). Overall, we identified 16 confirmatory studies, but with heterogeneous data. Psoriatic arthritis had a higher risk of positive antithyroperoxidase antibodies (TPOAb) (25%) compared to cutaneous psoriasis or control. There was an increased risk of thyroid dysfunction versus control, and hypothyroidism was the most frequent type of dysfunction (subclinical rather than clinical), among thyroid anomalies correlated with >2-year disease duration, peripheral > axial and polyarticular involvement. With a few exceptions, there was a female predominance. Hormonal imbalance included, most frequently, low thyroxine (T4) and/or triiodothyronine (T3) with normal thyroid stimulating hormone (TSH), followed by high TSH (only one study had higher total T3). The highest ratio of thyroid involvement concerning dermatologic subtypes was 59% for erythrodermic psoriasis. Most studies found no correlation between thyroid anomalies and psoriasis severity. Statistically significant odds ratios were as follows: hypothyroidism: 1.34–1.38; hyperthyroidism: 1.17–1.32 (fewer studies than hypo); ATD: 1.42–2.05; Hashimoto’s thyroiditis (HT): 1.47–2.09; Graves’ disease: 1.26–1.38 (fewer studies than HT). A total of 8 studies had inconsistent or no correlations, while the lowest rate of thyroid involvement was 8% (uncontrolled studies). Other data included 3 studies on patients with ATD looking for psoriasis, as well as 1 study on psoriasis and thyroid cancer. ICP was shown to potentially exacerbate prior ATD and psoriasis or to induce them both de novo (5 studies). At the case report level, data showed subacute thyroiditis due to biological medication (ustekinumab, adalimumab, infliximab). Thyroid involvement in patients with psoriasis thus remained puzzling. We observed significant data that confirmed a higher risk of identifying positive antibodies and/or thyroid dysfunction, especially hypothyroidism, in these subjects. Awareness will be necessary to improve overall outcomes. The exact profile of individuals diagnosed with psoriasis who should be screened by the endocrinology team is still a matter of debate, in terms of dermatological subtype, disease duration, activity, and other synchronous (especially autoimmune) conditions.

## 1. Introduction

Psoriasis, a complex chronic autoimmune multisystem disease with skin as its dominant manifestation, affects between 1% and 8% of adults worldwide [1,2]. While genetic predisposition is important, environmental factors, comorbidities and behavioral elements also matter [3]. The disorder has five major subtypes—plaque, inverse, guttate, pustular (PP) and erythrodermic (EP)—with the most frequent being chronic plaque psoriasis. This latter is characterized by erythematous, well-demarcated, indurated plaques with white-silvery thick scales. These can be either asymptomatic or pruritic, and typically involve the extensor surfaces, gluteal, and sacral areas. Additionally, specific sites may impose a higher burden on the quality of life of patients, such as palmo-plantar surfaces, the scalp, facial area, and the nail apparatus [4,5,6]. Psoriatic arthritis (PsA), an inflammatory polymorphic arthritis, occurs in up to 20–30% of individuals diagnosed with psoriasis. The majority of cases are identified after (or concurrently with) psoriasis vulgaris (PV). In about half of patients, PsA progresses to a destructive erosive disease with associated functional impairment [3,5,6,7]. Modern approaches to the treatment of psoriasis vary from molecular studies (to better understand pathogenic insights) to complex management in association with comorbidities’ assessment to seek better outcomes [6,8].

Pathogenic mechanisms involve a dysregulation of the innate and adaptive immune systems, primarily a T helper 1 cell and T helper 17 cell/interleukin-23 (IL)-mediated immune response, which may also involve IFN-γ (interferon), TNF-α (tumor necrosis factor), IL-17A, IL-12, and IL-23 [6,9]. Triggers, such as infections or local trauma, lead stressed keratinocytes to release molecules. These may include fragments of self DNA, self RNA, and antimicrobial peptides such as cathelidicin LL37, which stimulates plasmacytoid dendritic cells [6,10]. These cells, through their secretion of IFN-α, activate myeloid dendritic cells which migrate to lymph nodes and present this still unknown antigen to naïve T lymphocytes [6]. The myeloid dendritic cells promote differentiation to T helper 1, T helper 17 and T helper 22 subsets via IL-12 and IL-23 secretion [6,11,12]. Subsequent inflammatory cascades lead to keratinocyte hyper-proliferation with abnormal keratinization, excessive angiogenesis, induction of endothelial adhesion molecules, and a cellular infiltrate comprised of macrophages, dendritic cells and IL-17 secreting cells. This creates a feedback loop between keratinocytes and the immune cells that sustain and promote psoriasis plaque formation [13,14].

Psoriasis is associated with various comorbidities, including insulin resistance, metabolic syndrome, cardiovascular diseases, gastrointestinal diseases, and mental health disorders, imposing a great impact on overall quality of life [15,16,17,18,19]. Furthermore, individuals seem to be at greater risk of developing different autoimmune disorders like Crohn’s disease, vitiligo, celiac disease, ulcerative colitis, rheumatoid arthritis, type 1 diabetes mellitus, respiratory diseases, and autoimmune thyroid diseases (ATD), in addition to any of these or a single comorbidity [15,16,17,18,19].

ATD, with an estimated prevalence of 5% in general population, leads to two autoimmune disorders, situated at the end of the same spectrum: (1) chronic autoimmune (lymphocytic) Hashimoto’s thyroiditis (HT), with a higher risk for hypothyroidism due (albeit not exclusively) to thyroid blocking antibodies, namely antithyroperoxidase antibodies (TPOAb) and antithyroglobulin antibodies (TgAb), respectively; (2) Graves’ disease (GD), or Basedow–Graves’ disease, caused by thyroid-stimulating immunoglobulin or thyroid-stimulating hormone (TSH) receptor antibodies (TRAb), the sole human antibody with stimulating effects [20,21,22,23]. ATD is related to a dysregulation of the immune system, with lymphocytic infiltration of the thyroid gland and associated increased production of autoantibodies. The exact pathogenesis remains an open issue, but both environmental factors (infections, withdrawal of glucocorticoid therapy, stress, etc.) and genetic factors (genes associated with human leukocyte antigen (HLA) system, *AIRE* gene, or encoding genes for selenoproteins, etc.) are potentially involved [24,25,26].

### Aim

Our purpose was to overview clinical and pathogenic insights concerning psoriasis and thyroid comorbidities from a dual perspective (dermatologic and endocrine).

## 2. Methods

**Figure 1:**
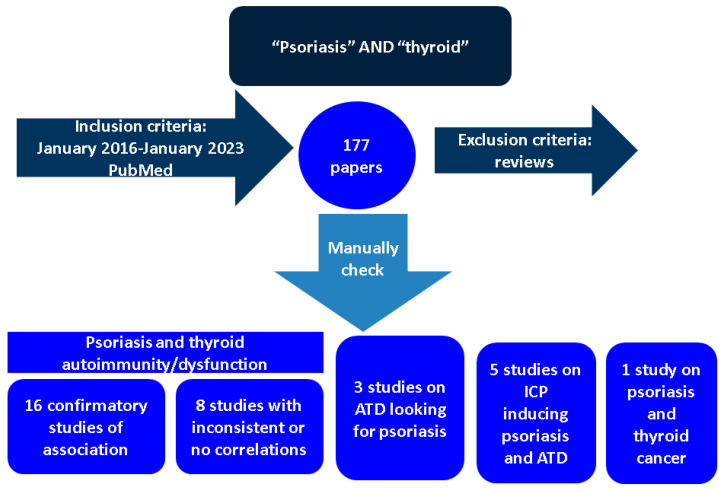
Flowchart of research according to our methodology (please see references below). (Abbreviations: ATD = autoimmune thyroid disease; ICP = immune checkpoint inhibitors).

This was a narrative review of literature published in English between January 2016 and January 2023. We included clinically relevant, original studies in humans, with different levels of statistical evidence, starting from two keywords used in PubMed research: “psoriasis” and “thyroid”. We identified 177 full-length papers and manually searched each of them in order to serve our purpose (Figure 1).

### 2.1. Thyroid Involvement (Hormonal Imbalance and Positive Autoimmunity) in Individuals with Psoriasis

#### 2.1.1. Confirmatory Data of Association

Positive correlations between psoriasis (including the subgroup with PsA) and ATD and/or thyroid hormonal anomalies were identified in different studies, aiming to address the hormonal imbalance, the autoimmune background, or both. Of note, HT diagnostic is typically sustained based on positive serum antibodies, while associated thyroid functions may be hypothyroidic, thyrotoxic, or normal, depending on disease evolution and applied therapy. GD is usually associated with hyperthyroidism at first diagnosis, but the copresence of HT may induce hypo- or euthyroidism. Additionally, any thyroid dysfunction may be at the clinical or subclinical levels. Moreover, abnormal thyroid hormone levels might not necessarily be related to an autoimmune background [27]. Knowing these dynamic aspects, a cross-sectional analysis may not capture the evolutionary aspects and complex inter-relationships between these skin and endocrine disorders.

The most important studies, having confirmatory profiles with regard to thyroid involvement, in patients known with psoriasis, according to our methodology, were as follows. First, a prospective study conducted by Vastarella et al. [28] analyzed the prevalence of HT in subjects confirmed with two types of psoriasis: PsA (N1 = 108) and cutaneous psoriasis (PsC) (N2 = 100). They showed that HT-associated subclinical hypothyroidism was more frequent in the PsA group and the ratio of positive TPOAb was increased in PsA versus PsC (13.9% versus 2%, *p* = 0.0018, respectively; 25.9 versus 9%, *p* = 0.018). Additionally, thyroid anomalies were more often found in subjects with PsA with established disease (≥2 years) than early disease (*p* < 0.05), and in those with peripheral involvement, when compared to axial PsA (85.7% versus 14.3%, *p* < 0.05). However, in PsC category, psoriasis severity was similar regardless of the copresence of ATD. A greater inflammatory state in patients with PsA, compared to patients without joint involvement, could possibly represent a factor with which to aid in identifying thyroid anomalies [29]. Another factor that could explain the different degrees of prevalence of HT was the female predominance in the PsA group, in contrast to the PsC group (52.7% versus 37%) [28]. Generally, women are prone to any type of ATD, with a 4 to 10 times higher risk than males [30].

In a prospective longitudinal study, Fallahi et al. [31] followed patients with PsA, without evidence of thyroid dysfunction (N = 97) versus control (N = 97), for 92 months. The PsA group developed TPOAb positivity (*p* < 0.014) and hypothyroidism (*p* < 0.05) (N = 97) more quickly than controls (N = 97), but this was not true of hyperthyroidism. PsA patients with subclinical hypothyroidism, compared to PsA individuals without any thyroid disorder, had a longer course of disease (18 ± 17 versus 9 ± 9 years; *p* = 0.005) and exhibited polyarticular involvement (*p* < 0.05). Logistic regression identified statistically significant risk factors for developing hypothyroidism in the PsA group, as follows: female gender, positive TPOAb, and a small thyroid volume at cervical ultrasound. No association was found between thyroid hormones and/or antibodies levels and PsA-associated disease activity/severity [31].

As previously shown by a Rotterdam study [32], a systematic review of 7 case-control studies and a meta-analysis of 4 studies confirmed a higher risk of ATDs in subjects with psoriasis [33]. The analysis revealed a link between psoriasis and thyroid hormones anomalies, as well (hypothyroidism (OR = 1.34; 95% CI 1.16–1.54)), and hyperthyroidism (OR = 1.17; 95% CI 1.03–1.32) [33].

A retrospective study by Du et al. [34] evaluated the relationship between different types of psoriasis and thyroid anomalies in 469 patients with PP, EP, PsA, and PV, versus 200 psoriasis-free controls (sex- and age-matched subjects). Individuals with EP had decreased levels of free triiodothyronine (T3) or free thyroxine (T4), with normal TSH (*χ*^2^ = 29.816, *p* < 0.001); patients with PP had decreased fT3 versus non-PP subtypes (*p* = 0.04); PsA patients had increased levels of TSH (*p* < 0.05). However, the levels of positive antibodies (TPOAb and TgAb) were similar between the studied subgroups and controls [34]. Another prospective study concerning different types of psoriasis included 63 patients with palmoplantar pustulosis and found a higher prevalence of thyroid disease in these individuals, compared to 34 subjects with PV (31.75% versus 13.51%; *p* = 0.0421) [35]. Another cross-sectional study on 102 persons suffering from palmoplantar pustulosis showed that comorbidities impacted the quality of life among them, with 16% suffering from ATD [36].

Another retrospective study by Namiki et al. [37] showed a higher rate of thyroid dysfunction in patients with generalized PP (GPP), when compared to PV and PsA (GPP versus PV, *p* = 0.0037; GPP versus PsA, *p* = 0.0348), with half of the thyroid anomalies being low T3 or T4 serum levels. The presence of thyroid dysfunction correlated with higher Psoriasis Area and Severity Index (PASI) scores of 21.0 ± 3.2 versus 13.5 ± 1.2 (Shapiro–Wilk test, *p* < 0.0001; respective Wilcoxon rank sum test, *p* = 0.0151) and increased C reactive protein (CRP) levels (5.56 ± 2.98 versus 0.73 ± 0.25 mg/dL; Shapiro–Wilk test *p* < 0.0001; Wilcoxon rank sum test *p* = 0.0069) [37]. However, the study indicated a higher prevalence of abnormal thyroid hormone profiles in men, as compared to women, which was opposed to most published data [28,30]. While no relationship was established between CRP levels and TSH, a negative correlation was observed between CRP and fT3, respectively, and fT4 (CRP versus TSH, r = -0.0504, *p* = 0.1777; CRP versus fT3, r = −0.4635, *p* = 0.0032; CRP versus fT4, r = 0.1242, *p* = 0.0181) [37].

Zheng et al. [38] published a retrospective study in 2020 on 201 patients with PV, PsA, GPP, and EP, along with 80 controls (individuals with noninflammatory skin conditions). The highest prevalence of thyroid dysfunction was found in the EP group (59.57%), followed by non-EP categories: 42.11% (GPP), 19.05% (PsA), and 18.99% (PV). The EP group was statistically significantly more affected than the PsA group (*p* < 0.001), but not the GPP group (*p* = 0.13). It was higher than the control group (*p* < 0.001) (GPP versus control (*p* = 0.005)). Two-thirds of the patients with psoriasis exhibited low levels of fT4, with normal TSH as the main abnormal hormonal finding. CRP levels were similar between psoriasis-positive subjects displaying thyroid hormone anomalies and those with normal thyroid profiles [38]. It bears mentioning that the general endocrine populations’ associations with various thyroid conditions do not typically associate anomalies with serum CRP levels except in subacute (viral) or acute (microbial) thyroiditis. That is why, from a strictly endocrine perspective, assessments of CRP add little value to our understanding of common mechanisms with psoriasis [39].

Wang et al. [40] conducted a large retrospective cohort study (National Health Insurance Research Database of Taiwan) including 162,842 individuals with psoriasis (PsA subgroup of 13,266 participants) with 1:1 sex- and age-matched controls (psoriasis-free). The studied population had an increased risk of developing thyroid hormonal imbalances, such as hyperthyroidism (aHR = 1.22, 95% CI 1.11–1.33), hypothyroidism (aHR = 1.38, 95% CI 1.23–1.56), ATD (aHR = 1.42, 95% CI 1.22–1.64), GD (aHR = 1.26, 95% CI 1.13–1.41), and HT (aHR = 1.47, 95% CI 1.18–1.82). The PsA group also showed a 1.44-fold increase in their risk for nontoxic goiter (95% CI 1.24–1.66), a 1.32-fold increased risk for hyperthyroidism (95% CI 1.07–1.65), a 2.05-fold increased risk for thyroiditis (95% CI 1.51–2.77), a 1.38-fold increased risk for GD (95% CI 1.07–1.79), and a 2.09-fold increased risk for HT (95% CI 1.34–3.24) [40].

Similarly, Liu et al. [41] analyzed patients with psoriasis and incident thyroid morbidity (US population-based study), enrolling 15,091 adults (National Health and Nutrition Examination Survey between 2009 and 2014). They confirmated increased risks for thyroid dysfunction (aOR = 1.607; 95% CI 1.011–2.554), mostly affecting those between 40 and 59 years (aOR = 2.667; 95% CI 1.376–5.168) [41]. Kiguradze et al. [42] published a large, cross-sectional cohort study (Northwestern Medicine Enterprise Data Warehouse) on 9654 individuals with psoriasis and 1745 patients with HT; the association between these two disorders was confirmed after adjusting for confounding variables such as gender, age, PsA, and use of systemic antipsoriatic agents (OR = 2.49; 95% CI 1.79–3.48; *p* < 0.0001) [42]. Valdulga et al. showed that HT prevalence was higher than controls (N = 60 patients with psoriasis versus 60 gender- and age-matched controls: 21.6% versus 6.6% (*p* = 0.03)). Among subjects with psoriasis, women were more frequently affected by HT (*p* = 0.002), and logistic regression confirmed that plaque psoriasis was the single independent variable associated with HT [43].

A meta-analysis from 2022, performed by Zhang et al. [44] and involving 253,313 subjects with psoriasis and 1,376,533 controls, showed an increased prevalence of ATDs in psoriasis group versus control (OR = 1.76, 95% CI 1.35–2.28, *p* < 0.01). When analyzing the prevalence of a specific ATD, HT was significantly more prevalent in patients with psoriasis than controls (OR = 1.88, 95% CI 1.50–2.35, *p* < 0.01), but not GD [44].

We identified one study on individuals with psoriasis comparing late onset (after the age of 40) with early onset (before the age of 40) cases (278, respectively, 62 individuals). A higher risk of autoimmune thyroiditis was revealed in first group (adjusted OR = 5.05; 95% CI, 1.62–15.7) [45].

Overall, associations between psoriasis, as a general condition as well as its different subtypes, and thyroid disorders, in terms of abnormal thyroid hormone levels and/or thyroid antibodies, were confirmed by these mentioned studies. The results were heterogenous [40,41,42,43]. The extent of statistical relevance varied with study design, enrolled population, specific endocrine assessments, and dermatologic evaluation (types of psoriasis, disease duration, score of activity/severity, PsA association, etc.). The duration of psoriasis disease increased the risk of detecting thyroid abnormalities. The most frequent associations were observed with HT, not GD, while the most frequent hormonal imbalance seemed to be hypothyrodism (sublinically, rather than clinically, manifested). The severity of psoriasis did not seem to correlate with the presence of thyroid dysfunction and/or autoimmunity in most studies [29,31], though some exceptions were reported [37,46] (Table 1).

#### 2.1.2. Studies with Inconsistent Correlations between Psoriasis and Thyroid Anomalies

A small case-control study evaluated associations between psoriasis and HT (N = 56 versus 54 controls); similar TSH and fT4 levels were found between the two studied groups. However, higher levels of prevalence of TPOAb and TgAb were observed in the psoriasis group than in the control group. There was also an increased rate of ultrasound findings suggesting ATDs, such as hypo-echogenicity (30.4% versus 9.3%, *p* = 0.02), high vascularity (35.7% versus 5.6%, *p* = 0.001), and pseudo-nodularity (16.1% versus 0%, *p* = 0.002). Severity of disease (PASI score) was not correlated with TPOAb or TgAb positivity [47], as in prior mentioned studies [30,31].

Hansen et al. [48] enrolled a previously-studied population from the Danish General Suburban Population Study [49]. Individuals with psoriasis (N = 1127) were matched (1:5) with healthy controls with regards to gender, age, body mass index, and smoking status. ATD and TPOAb were similar between the groups. Individuals with psoriasis had a higher total T3 (1.69 ± 0.32 versus 1.72 ± 0.33 nmol/L; *p* = 0.01), but similar levels of TSH and free T4. The exact mechanism behind high total T3 levels, along with normal TSH and total T4, in the studied population was not clearly understood [48]. No correlation was confirmed between psoriasis and thyroid involvement by Lai et al. [50], who analyzed a random population sample of 5560 responders from the U.S. National Health and Nutrition Examination Survey database between 2011 and 2012. Adjusting for confounding factors, such as body mass index, age, gender, smoking habits, and alcohol consumption, yielded results similar to several previous studies [50,51].

Vassilatou et al. [52] examined the prevalence of ATD in subjects with psoriasis (N = 114) in a prospective study (N = 286 age- and body mass index-matched controls, without a history of psoriasis, from areas with sufficient iodine intake). After defining HT as TPOAb and TgAb serum titers over 34 IU/mL and 115 IU/mL, respectively, and evaluating PASI scores, TSH, T3, T4, fT4, and antibody levels were similar between the groups. However, the authors confirmed female predominance by identifying an increased prevalence of HT in females in the control group (14.7% versus 4.9%), but not in the psoriasis groups (10.5% versus 9.6%) [52].

A study focusing on quality of life in patients with psoriasis (N = 74) showed a weak correlation with the presence of thyroid diseases (affecting 6.75% of them) [53]. Another retrospective, observational study showed that patients with plaque psoriasis with thyroid involvement were similar in age, gender, disease severity, and duration to those with normal thyroid profiles. In total, 10% of the entire cohort (N = 290) experienced a thyroid dysfunction (defined as a ≥10% variation in normal thyroid hormone values), while 13.5% of individuals with psoriasis had positive serum TPOAb [54]. Another small study (without a control group) showed that, among 48 patients with palmoplantar pustulosis, 12% had antibody-based thyroiditis [55]. The Mayo Clinic published a retrospective, uncontrolled study on 215 persons with palmoplantar pustulosis, and identified 18 subjects (8%) with thyroid diseases. This was less than expected, according to Olazagasti et al. [56] (Table 2).

### 2.2. Pathogenic Elements Involving Psoriasis and Thyroid Comorbidities

#### 2.2.1. Traditional Pathogenic Frame

Thyroid hormones are critical regulators of development. They function at various levels, including the digestive system, cardiac and skeletal muscle, and brain; they also affect energy metabolism and overall homeostasis [57,58]. Skin is involved in thyroid hormone activity, signaling which hormones exert their roles through genomic mechanisms (i.e., by binding nuclear thyroid hormone receptors) as well as non-genomic pathways, involving cellular proteins such as membrane integrin, αvβ3, etc. [57,58,59]. Thyroid hormones are implicated in fetal epidermal differentiation, barrier formation, hair growth, keratinocyte proliferation, and modulation of keratin gene expression [57,58,59]. They induce keratinocyte hyper-proliferation through epidermal growth factor (EGF). Data have shown that antithyroid medications for hyperthyroidism (for example, propylthiouracil) exhibit antiproliferative effects, with beneficial effects on psoriasis plaques [60,61].

Among the targets of T4 and T3, K6, K16, and K17 are connected with psoriasis pathogenic loops, while K1 and K10 are displaced in spinous and cornified layer [58,59,62]. Murine studies on thyroid hormone receptors in mutant mice (lacking TRα1 and TRβ isoforms) showed markedly reduced keratinocyte proliferations, increased activations of p65/NF-κB pathways, and STAT3 phosphorylation—which caused a high expression of pro-inflammatory cytokines and chemokines [58,63].

T-helper 1 lymphocyte dominant response, observed in psoriasis, determines a pro-inflammatory milieu, typically comprised of IFN-γ, TNF-α, and chemokines such as CXCL10 (a chemoattractant for neutrophils found in active psoriasis plaques) [64]. ATDs share a Th1 immune-mediated response with IFN-γ and IFN-γ dependent chemokines like chemokine (C-X-C motif) ligand (CXCL)10, involved in the pathogenesis of both GD and HT [5,64,65]. IL-17 of the IL-23/Th17 axis, as seen in psoriasis, plays an important role in ATD, indicating another potential level of connection between the two conditions [42,43,66]. A shift from a Th-1 to a Th-2 immune response through monocyte chemoattractant protein-1 (CCL2) and macrophage-derived chemokine CCL22 has been described in both PsA and GD [58,67,68].

#### 2.2.2. Recent Pathogenic Landscape

The latest data concern new pathogenic pathways to potentially connect psoriasis to different anomalies at the thyroid level, either through direct links or indirect associations with other conditions, especially those with a higher risk for developing both skin and thyroid diseases of the autoimmune type [69,70,71,72,73,74].

Jiang Y. et al. published a study in 2022 regarding the Psoriasis susceptibility 1 candidate 1 (*PSORS1C1*) gene, which has been associated with various autoimmune conditions, including rheumatoid arthritis, ankylosing spondylitis, and systemic lupus erythematosus. This case-control study involved 1065 patients (Chinese Han participants) with ATD and 943 healthy controls, and analyzed 4 single nucleotide polymorphisms (SNPs): rs3130983, rs3778638, rs3815087, and rs4959053. They determined that rs3778638 genotypes were statically significant different from ATD and control (*p* = 0.046), but the rs3778638 genotype was only correlated with GD (*p* = 0.039), not with HT (*p* = 0.141) [75]. Tumor necrosis factor α-induced protein 3 (*TNFAIP3*) gene was recently incriminated in a large spectrum of autoimmune disorders, including ATD and psoriasis [76].

Another common pathogenic mechanism concerns thyroid hormone signaling, potentially involved in the microRNA-ome underlying psoriatic skin [77]. Anomalies of apoptosis affecting keratinocyte proliferation in psoriasis have been described in ATD, as well [78]. Defects of apoptotic pathways might represent a link to metabolic syndrome in PV and hypothyroidism [79].

A recent hypothesis suggested that viral infections in pregnant females could trigger autoimmune conditions early in life, including type 1 diabetes mellitus, HT, and psoriasis [80]. Another clinical circumstance for developing both ATD and psoriasis was found in HIV-positive and hepatitis C-positive patients to whom prolonged survival was recently registered due to advance of antiviral drugs. A higher risk of developing different autoimmune disorders was identified [81,82]. Globally, one-third of adults presenting common variable immunodeficiencies are admitted for autoimmune comorbidities, including psoriasis and thyroiditis of different kinds [83]. Another clinical entity that may be associated with a higher risk of psoriasis and HT is idiopathic retroperitoneal fibrosis, an immune-mediated condition involving a large frame of chemokines (e.g., CXCL12 and CCL11) and cytokines (e.g., IL-6, IL-12, and IL-13) [84]. Another clinical example is primary biliary cholangitis; a study from 2021 (N = 1554 patients with this condition) showed that ATD coexisted in 10.6% of cases, while 1.5% had psoriasis [85].

Moreover, both HT and psoriasis have been listed as autoimmune complications triggered by infection with *Helicobacter pylori*, in association with positive gastric autoimmunity [86]. Additionally, the prescription of proton pump inhibitors could exacerbate autoimmunity (including HT and psoriasis) under certain circumstances [87].

Another potential iatrogenic component relates to dipeptidyl peptidase-4 inhibitors (DPP4is), prescribed for inflammatory diseases due to their inhibitory effects on cytokine production and T cell proliferation. A population-based study on 283 individuals treated with these agents, versus 5660 controls, showed a higher prevalence of psoriasis (2.5% versus 1.2%; OR= 2.12; 95% CI 0.99–4.66; *p* = 0.05), respective to HT (16.6% versus 12.6%; OR = 1.38; 95% CI 1.00–1.91; *p* = 0.049) [88].

A multimodal approach was proposed, involving thyroid hormones and vitamin D as players in psoriasis lesions progression [89]. One small study on 30 patients with psoriasis and 30 healthy controls showed a higher serum TSH value in the psoriasis group (*p* < 0.05), but with intra-normal TSH variations and a negative correlation between serum 25-hydroxyvitamin D and PASI [90]. Another endocrine component of psoriasis and disorders associated with an abnormal thyroid profile, like thyroid eye disease (in GD), potentially involves insulin-like growth factor (IGF) axis [91].

**Figure 2:**
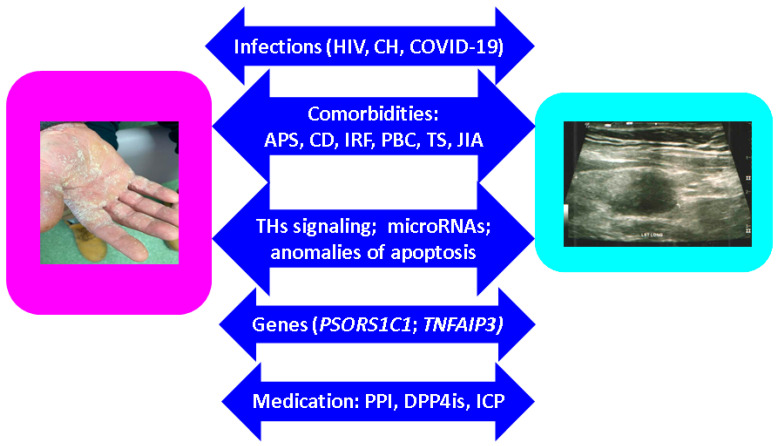
Potential pathogenic connections or pathologic circumstantial events involving psoriasis and ATD [69,70,71,72,73,74,75,76,77,78,79,80,81,82,83,84,85,86,87,88,89,90,91,92,93,94,95]. Abbreviations: CH = C hepatitis; APS = autoimmune poly-glandular syndrome; CD = celiac disease; IRF= idiopathic retroperitoneal fibrosis; PBC = primary biliary cholangitis; TS = Turner syndrome; TH = thyroid hormone; PPI= proton pump inhibitors; DPP4is = ipeptidyl peptidase-4 inhibitors; ICP = immune checkpoint inhibitors; JIA = juvenile idiopathic arthritis. Capture of the left: palmar surface involvement due to psoriasis; on the right: thyroid ultrasound with hypoechoic pattern, suggesting thyroiditis.

One of the most recent pathogenic factors is represented by COVID-19 infection, which seems to trigger various panel of single or poly-autoimmunity, potentially as part of long COVID-19 syndrome [92,93,94]. We identified a single case to highlight this combination: a previously healthy teenager with negative family history for autoimmunity who developed GD and PV after infection with COVID-19 [95]. Further data are expected to highlight autoimmunity following COVID-19 (Figure 2).

Of note, major histocompatibility complex (MHC) loci have been reported in relation with a myriad of autoimmune disorders, including many at the skin and thyroid levels. Antigen presentation by MHC-II is related to the immune response, including self-tolerance. Anomalies of MHC are connected to triggering autoimmune responses, involving T cells at many levels, as well as immune recognition, comprising both MHC-I and MHC-II. For instance, genetic susceptibility to PsA includes, among others, MHC-I-associated gene polymorphisms like *IL12B*, *TYK2*, etc., while haplotypes such as *DR3-DQ2* and *DR4-DQ8* are prone to autoimmune thyroiditis [96,97,98,99]. On the other hand, another modern field of common pathogenic interest for many autoimmune diseases, including psoriasis and ATD, bears mentioning: gut microbiota. Anomalies of intestinal metabolites or abnormal interactions between intestinal microorganisms and human host systems might be a cornerstone factor contributing to autoimmune responses [100]. In many skin diseases with autoimmune backgrounds, dual interplays between the immune system, which modulates normal dermatological processes, and intestinal microorganisms (underlying dysbiosis) have been reported [101,102]. Both microbiomes and macrobiomes have been described in terms of their relationships to developing psoriasis [103]. Moreover, Chao1 (which is the index of microflora richness) has been found at increased levels in HT and decreased levels in GD [100,104]. Additionally, T3, by activating its receptor α1 at the intestinal level, represents a contribution to epithelial homeostasis, while metabolite-derived short-chain fatty acids modulate thyroid function [105,106].

### 2.3. Identifying Psoriasis in Patients with Previous Positive Thyroid Autoimmunity

Skin and hair conditions have been reported in patients suffering from hypo- or hyperthyroidism of different etiologies, but most reports have been in subjects with ATD, as a single endocrine complication or as part of autoimmune polyglandular syndromes (APS) [107,108,109]. Vitiligo and alopecia have been identified in antibody-related circumstances similar to psoriasis, including those with pediatric onset [107,108,109]. The panel of endocrine conditions in ATD-positive subjects also includes primary ovarian failure, autoimmune hypoparathyroidism, hypophysitis, premature ovarian failure, Addison’s disease, and type 1 diabetes mellitus, among others. [110,111,112]. Non-endocrine autoimmune features include, among others, lupus, dermatomyositis, gastritis, hepatitis, and colitis. [100,101,102,103,104,105,106,107,108,109,110,111,112,113,114,115]. For instance, one recent study, from 2022, on 116 patients with Addison’s disease, showed that 74% of them had at least one relative confirmed with an autoimmune entity (N = 221 relatives with 257 diseases); among these, 100 individuals were identified with HT, and 15 were determined to have psoriasis [116].

With regard to assessing psoriasis in endocrine patients, we identified 3 studies, according to our methodology. Fallahi et al. [117] conducted a prospective study on 3069 individuals with autoimmune thyroiditis and found a higher prevalence of PsA in these patients (*p* < 0.0180) when compared to controls. However, no statistically significant evidence was attained for the PsC subgroup (*p* = 0.6237) [117]. Kelada et al. retrospectively studied a population diagnosed with thyroid eye disease (GD-associated autoimmune orbitopathy) between 2011 and 2019 (N = 267). Of these, 13.9% displayed non-thyroid autoimmune comorbidities, associated with a more severe/active eye presentation, and 3% of subjects had psoriasis [118]. Takir et al. [119] examined, in a cross-sectional study, 300 persons with thyroid diseases (N1 = 173 with autoimmune disorders, N2 = 127 with non-autoimmune conditions) versus 100 healthy controls. Psoriasis was identified as statistically significantly more frequent in N1 than N2 (*p* = 0.001), suggesting that patients under endocrine surveillance were more likely to have psoriasis if their thyroid condition were autoimmune [119] (Table 3).

## 3. Discussions

### 3.1. Thyroid Cancer in Patients with Psoriasis

Patients with psoriasis are at higher risk of some cancers, and some safety concerns arise in relation to thyroid cancer, as well. However, the fact that patients with psoriasis display an increased risk of thyroid malignancy remains controversial [120,121]. As mentioned, one single study identified a higher risk of nontoxic goiter in PsA [40]. Of note, 5% of the general population (depending on age) has a thyroid nodule, while thyroid cancer represents the most frequent endocrine neoplasia, with an age-dependent prevalence of almost 5% of thyroid nodules [122,123].

We identified only one study which examined thyroid cancer in individuals with psoriasis. This was a nested case-control study (Korean National Health Insurance Service-Health Screening Cohort) that included individuals 40 years and older (N = 6822 subjects with thyroid cancer versus 27,288 controls). A previous history of psoriasis was similar between studied groups (OR = 1.02; 95% CI, 0.85–1.22). The subgroup without hypothyroidism had a higher rate of thyroid malignancy associated with psoriasis (overlap-weighted OR = 1.29; 95% CI 1.06–1.57, *p* = 0.012) while those with hypothyroidism showed a low rate (overlap-weighted OR = 0.59; 95% CI 0.37–0.96, *p* = 0.034); no other correlations were identified [121] (Table 4).

Based on these findings, we concluded that we did not have sufficient data to support an association between thyroid cancer and psoriasis.

### 3.2. Psoriasis and Thyroiditis among Immune Side Effect of Anti-Cancer Drugs

Medication used in psoriasis treatment could contribute to the development of immune and autoimmune events with concomitant anticancer drugs. On the other hand, both ATD and psoriasis have been incidentally reported in oncologic patients who developed immune/autoimmune side effects to modern categories of immune checkpoint inhibitors [124,125]. Pre-existent autoimmune conditions increase the risk of developing immune side effects while being treated with anticancer medication [126]. Several studies have observed exacerbation of psoriasis and ATD in oncologic patients [127,128,129,130,131] (Table 5).

The largest study, as of 2016, on ipilimumab treatment for melanoma in patients with preexistent autoimmune conditions, showed that one-third of the subjects suffered an exacerbation of prior comorbidities; one-third of these exacerbations were reversible upon glucocorticoid exposure [131].

Studies published within the last few years confirmed that previous psoriasis or ATD may worsen in response to immune checkpoint blockade. In cases with thyroiditis, glucocorticoid therapy and antithyroid medication for hyperthyroidism or, alternatively, substitution with levothyroxine for hypothyroidism, is required, sometimes for the remainder of a patient’s life [129]. A meta-analysis from 2021 on randomized, placebo-controlled studies, including oncologic patients under immune checkpoint inhibitors (N = 5560), showed an incidental rate of thyroiditis (0.86%), and identified one new case with psoriasis [128].

### 3.3. Subacute Thyroiditis in Patients Treated for Psoriasis

Anti-psoriasis medications, as contributors to thyroid anomalies, include anti-TNF-α drugs; their contributions remain a matter of debate [132]. Subacute thyroiditis, despite being a classically viral condition, has been reported in circumstances involving an abnormal cytokine profile [133]. Biological medications for skin conditions might play a role in triggering flare-ups of colloidal pools filled with thyroid hormones T3 and T4, as seen in thyroiditis-associated thyrotoxicosis [134,135].

For instance, there was a case of a 32-year-old male with confirmed thyrotoxicosis being treated with ustekinumab (monoclonal antibody against IL-12/23). He relapsed twice after reinitiation of the drug [136] Another case (published in 2021) introduced a 71-year-old male patient with PsA who was treated with secukinumab (IL-17A inhibitor), which was later switched to adalimumab (TNF-α inhibitor), while developing subacute thyroiditis. Therapy with prednisolone was necessary. Consecutive treatment with ixekizumab (IL-17A inhibitor) controlled PsA and did not induce a relapse of thyroiditis [137]. Another case of adalimumab-associated subacute thyroiditis was reported in 2017 [138]. Cytomegalovirus-induced subacute thyroiditis was reported in 2016 in a patient with PsA treated with infliximab [139].

### 3.4. Pediatric Population

As mentioned, most of the data included adult patients. However, APS can be associated with HT, especially type 3, and psoriasis has been reported, extremely rarely, in adults and in children [140,141].

Moreover, results from the International Pharma-Child Registry confirmed that both psoriasis and ATD were among the most frequent autoimmune disorders in populations diagnosed with juvenile idiopathic arthritis. Positive familial autoimmune diseases have been determined to be a risk factor for developing this type of arthritis [142]. Concerning the burden of autoimmune comorbidities in 79 individuals diagnosed with juvenile idiopathic arthritis (aged between 0 and 21 years), a rate of 10% (N = 8) was identified with ATD, while approximatively 4% had psoriasis [143]. Cumulative incidence of ATD was 36%, with mean age at diagnosis of 13.2 years. First-degree relatives were more affected by autoimmune comorbidities than second-degree relatives (16.7% versus 11%) [143].

### 3.5. Interventional Considerations

Interventional studies addressing psoriasis and ATD remain an open issue. Some nutraceutical supplements could improve both psoriasis and HT [144]. Among these, vitamin D supplementation and omega 3 fatty acid supplementation could potentially reduce the burden of autoimmune diseases, but only upon exposure to certain doses and only for a limited period of time [145]. It has not been determined whether or not hypo- or hyperthyroidism in subjects with psoriasis improves skin condition. As has been pointed out, individuals with the most severe psoriasis cases may not necessarily be those displaying a pathological profile, with respect to the thyroid gland.

Gluten-free diets have yet to be proven useful in ameliorating psoriasis and ATD, as suggested by some authors. Based on current data, unless celiac disease is co-present, this routine recommendation is not supported [146]. Additionally, vegan and vegetarian habits might trigger phytophotodermatitis; thus, diet could play an important role in modulating thyroid profiles and in psoriasis therapies [147]. The association with celiac disease is not rare; one retrospective study on 749 patients with this digestive condition showed a prevalence of 19.9% for ATD, 14.7% for hypothyroidism, and 2.7% for psoriasis [148]. Additionally, a case-controlled study on 341 individuals with celiac disease showed that 26.6% of them had at least one autoimmune disease (autoimmune thyroiditis, 48%; psoriasis, 17%) [149]. Another case-controlled study on 255 persons with celiac disease (versus 250 controls) showed that 35.2% of them had autoimmune disorders. HT was the most frequent comorbidity (24.3% versus 10%), while the second most prevalent was psoriasis (4.3% versus 1.6%) [150]. Another endocrine condition that can predispose someone to all three mentioned autoimmune diseases is Turner syndrome. In patients suffering from Turner syndrome, lifelong surveillance—including monitoring for autoimmune complications—is necessary [151,152].

Future studies will likely continue the search for an ideal drug to concomitantly target disorders like psoriasis and ATD.

### 3.6. From Today to Tomorrow

**Figure 3:**
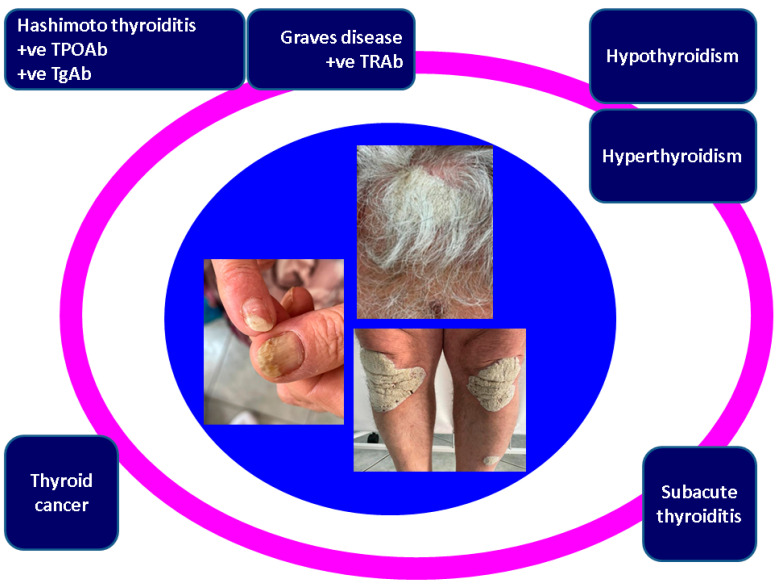
Qualitative analysis of thyroid involvement in psoriasis, according to our methodology. Abbreviations: TPOAb = antithyroperoxidase antibodies; TgAb = antithyroglobulin antibodies. Central captures: nail psoriasis (subungual hyperkeratosis, nail plate thickening and the “oil drop” sign); bilateral plaques on the lower extremities with thick scales; diffuse scalp involvement.

Strategically searching for thyroid anomalies in patients confirmed with psoriasis, either on clinical or molecular levels or using genetic findings, also remains an open issue. The exact subgroup of individuals that should be candidates for strategic endocrine assessments has not been determined. The minimum panel of thyroid assays includes: TSH, freeT4 (perhaps also freeT3), and TPOAb (perhaps also TgAb). We followed 4 clusters of conditions: thyroid dysfunction, autoimmunity, thyroid cancer, and subacute thyroiditis. The fact that psoriasis and ATD are related to the immune-based side effects of modern anticancer drugs, namely immune checkpoint inhibitors, is a new piece of information in this field (Figure 3).

**Figure 4:**
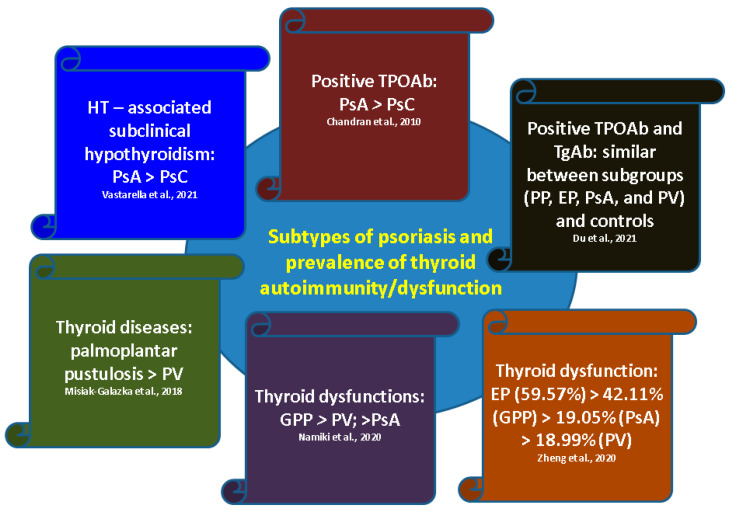
Analysis of specific psoriasis subtypes and thyroid anomalies according to our methodology (please see references in main text above). References [28,29,34,35,37,38] are pointed out in each box. Abbreviations: EP = erythrodermic psoriasis; PsA = psoriatic arthritis; PsC = cutaneous psoriasis; PP = pustular psoriasis; PV = psoriasis vulgaris; GPP = generalized PP; TPOAb= antithyroperoxidase antibodies; TgAb = antithyroglobulin antibodies; HT = Hashimoto’s thyroiditis.

To our knowledge, this was one the most complex analyses of published studies concerning a dermatological and endocrine dual perspective. Overall, we identified 16 confirmatory studies, but with heterogeneous data. PsA had a higher risk of positive TPOAb (25%) versus PsC or control, as well as an increased risk of thyroid dysfunction versus control. Hypothyroidism was the most frequent type of dysfunction (subclinical rather than clinical). Thyroid anomalies were correlated with >2-year disease duration, peripheral > axial and polyarticular involvement. With a few exceptions, female predominance was observed. Hormonal imbalances included, most frequently, low T4 and/or T3 with normal TSH, followed by high TSH; only one study observed higher total T3. The highest ratio of thyroid involvement in dermatologic subtypes was 59% for EP. An analysis of specific psoriasis subtypes and associated thyroid anomalies, according to prior mentioned studies, can be found in Figure 4.

Most studies found no correlation between thyroid anomalies and psoriasis severity. Statistically significant ORs for hypothyroidism: 1.34–1.38; hyperthyroidism: 1.17–1.32 (fewer studies than hypothyroidism); ATD: 1.42–2.05; HT: 1.47–2.09; GD: 1.26–1.38 (fewer studies than HT). Additionally, 8 studies had inconsistent or no correlations or weak statistical power concerning associations between thyroid autoimmunity or dysfunction and psoriasis. The lowest rate of thyroid involvement was 8% (uncontrolled studies). Other data included: 3 studies on patients with thyroid autoimmune conditions looking for psoriasis and one study on psoriasis and thyroid cancer. ICP was shown to possibly exacerbate prior ATD and psoriasis or to induce them both de novo (5 studies). At the case report level, studies examined subacute thyroiditis due to biological medication (ustekinumab, adalimumab, infliximab). Further well-designed, longitudinal controlled studies are necessary.

## 4. Conclusions

Thyroid involvement in patients with psoriasis remains an open question. However, observed significant data that confirmed a higher risk positive antibodies and/or thyroid dysfunction, especially hypothyroidism, in subjects with psoriasis. Further study, and greater awareness, is necessary to improve overall outcomes for patients. Debate continues concerning the exact profile of individuals, diagnosed with psoriasis, who should undergo endocrinological screening. Uncertainty exists regarding dermatological subtypes, disease duration and activity, and other synchronous (especially autoimmune) conditions.

## Figures and Tables

**Table 1:** Studies (confirmatory associations) aiming to analyze thyroid profile in patients with psoriasis (from 2016 to the most recent, at the time of this writing) [17,28,31,33,34,35,36,37,38,40,41,42,43,44,45,46].

Authors/ Year of Publication Reference	Type of Study	Studied Population	Results
Khan 2016 [33]	Prospective cohort study (Rotterdam studyMeta-analysis) (4 studies) Systematic review (7 studies)	Rotterdam study:	TPOAb positivity 1077 (13.1%) Association between psoriasis and: TPOAb positivity: OR = 1.71 (CI: 1.27–2.31) ATD: OR = 1.25 (CI: 1.14–1.37) hypothyroidism: OR = 1.34 (CI: 1.16–1.54) hyperthyroidism: OR = 1.17 (CI: 1.03–1.32) for hyperthyroidism
		N = 8.214	
		mean age: 62.3 ± 8.4 y	
Theodorakopoulou 2016 [45]	Cross-sectional study	Patients with psoriasis:	Autoimmune thyroiditis N1 < N2: OR = 5.05; 95% CI 1.62–15.7
		N1 = 278 early onset (<40 y)	
		N2 = 62 late onset (>40 y)	
Kiguradze 2017 [42]	Cross-sectional study	N1 = 9654 individuals with psoriasis	Association between psoriasis and HT: OR = 2.49; 95% CI 1.79–3.48, *p* < 0.0001
		N2 = 1745 patients with HT	
Trattner 2017 [36]	Cross-sectional study		N = 102 patients with palmoplantar pustulosis
Fallahi 2017 [31]	Prospective study	N1 = 97 PsA	PsA more frequent vs. controls: positive TPOAb (*p* < 0.014) hypothyroidism (*p* < 0.05)
		mean age: 56 ± 12 y	
		N2 = 97 controls	
		mean age: 57 ± 11 y	
Misiak-Galazka 2018 [35]	Prospective study	N1 = patients with palmoplantar pustulosis	Thyroid disease: 31.75% vs. 13.51%; *p* = 0.0421
		N2 = 34 PV	
Namiki 2019 [37]	Retrospective study	N1 = 51 PV	Highest prevalence of thyroid dysfunction in GPP group (45% GPP vs. 13% PsA, respective 8% PV)
		mean age: 52.86 ± 21.0 y	
		N2 = 23 PsA	
		mean age: 46.7 ± 15.86 y	
		N3 = 11 GPP	
		mean age: 63.73 ± 11.63 y	
Wang 2019 [40]	Retrospective population-based cohort study	N1 = 149,576 PV	PV patients at risk of developing: hyperthyroidism aHR = 1.22, 95% CI 1.11–1.33 hypothyroidism aHR = 1.38, 95% CI 1.23–1.56 ATD aHR = 1.42, 95% CI 1.22–1.64 GD aHR = 1.26, 95% CI 1.13–1.41 HT aHR = 1.47, 95% CI 1.18–1.82
		mean age: 45.11 ± 20.09 y	
		N2 = 162,842 control group	
		mean age: 44.95 ± 19.91 y	
Vashist 2020 [17]	Pilot study	N = 80 patients with psoriasis	hypo and hyperthyroidism: 8.8% positive TPOAb: 5% positive TgAb: 1.3%
		age between 13 and 75 y	
Zheng 2020 [38]	Retrospective study	N1 = 74 PV	Highest prevalence of TD in EP (59.57%) followed by GPP (42.11%), PsA (19.05%), PV (18.99%)
		mean age: 56.12 ± 14.05 y N2 = 42 PsA	
		mean age: 53.79 ± 11.43 y	
		N3 = 38 GPP	
		mean age: 46.16 ± 17.69 y	
		N4 = 47 EP	
		mean age: 57.51 ± 15.20 y	
		N5 = 80 control group	
		mean age 56.78 ± 15.48 y	
Vastarella 2021 [28]	Prospective study	N = 208	PsA vs. PsC: subclinical hypothyroidism + positive TPOAb: 13.9% vs. 2% (*p* = 0.0018) positive TPOAb: 25.9 vs. 9% (*p* = 0.018)
		N1 = 108 PsA	
		mean age: 39.9 ± 10.8 y	
		N2 = 100 PsC	
		mean age: 50.1 ± 11.7 y	
Du 2021 [34]	Retrospective study	N1 = 300 PV	fT3 lower in PP group vs. PV/EP groups (*p* = 0.04) fT4 lower in EP group vs. PV group (*p* < 0.001), vs. PP (*p* = 0.019), and vs. PsA group (*p* < 0.001) TSH higher in PsA then in EP group (*p* = 0.049) similar levels of TPOAb and TgAb among the 4 subgroups (*p* > 0.05)
		mean age: 47.8 ± 15.5 y	
		N2 = 60 PP	
		mean age: 46.6 ± 18.6 y	
		N3 = 54 EP	
		mean age: 51.8 ± 15.8 y	
		N4 = 54 PsA	
		mean age: 47.4 ± 13.1 y	
		N5 = 200 controls	
Liu 2022 [41]	Population based cohort study		N = 15.091
Valdulga 2022 [43]	Cross-sectional observational study	N1 = 60 patients with psoriasis	HT prevalence: 21.6 vs. 6.6% (*p* = 0.002)|
		N2 = 60 controls	
Zhang 2022 [44]	Meta-analysis (11 studies)	N1= 253.313 PV	PV has: higher prevalence of ATD: OR = 1.76, 95% CI 1.35 to 2.28, Z = 4.25 (*p* < 0.01) higher rate of TgAb: OR = 1.98, 95% CI 1.27 to 3.10, Z = 3.00 (*p* < 0.01) higher rate of TPOAb: OR = 2.15, 95% CI 1.31 to 3.52, Z = 3.05 (*p* < 0.01)
		N2= 1.376.533 controls	
Yumnam 2022 [46]	Hospital-Based, Cross-Sectional Study	N = 111 patients with psoriasis	Thyroid dysfunction associated with a severe form of psoriasis versus mild psoriasis (61.9% vs. 38.1%)

Abbreviations: aHR = adjusted Hazard Ratio; aOR = adjusted Odds Ratio; ATD = autoimmune thyroid disease; CI = confidence interval; EP = erythrodermic psoriasis; fT3 = free triiodothyronine; fT4 = free thyroxine; HT = Hashimoto’s thyroiditis; N = number of patients; OR = odds ratio; PP = pustular psoriasis; GPP = generalized PP; PV = psoriasis vulgaris; PsC = cutaneous psoriasis; PsA = psoriatic arthritis; vs. = versus; years = y; TPOA = antithyroperoxidase antibodies; TgAb = antithyroglobulin antibodies; TSH = Thyroid Stimulating Hormone.

**Table 2:** Studies (non-confirmatory associations) aiming to analyze thyroid profile in patients with psoriasis (from 2016 to the most recent, as of this writing) [47,48,50,52,53,54,55,56].

Authors/ Year of Publication Reference	Type of Study	Studied Population	Results
Lai 2016 [50]	Population-based study	N = 5560 responders from 2011–2012 U.S. National Health and Nutrition Examination Survey database	No correlation between psoriasis and thyroid involvement
Olazagasti 2017 [56]	Retrospective study	N = 215 patients with palmoplantar pustulosis	thyroid diseases: 8%
Vassilatou 2017 [52]	Case-control study	N1 = 114 patients with psoriasis	Psoriasis group versus controls: similar TSH, T3, T4 and free T4
		N2 = 286 controls	
Aldrisi 2019 [47]	Case-control study	N1 = 56 PV group	PV versus controls: similar TSH, FT4 TPOAb positivity: OR = 3.2 (1.08–9.82), *p* = 0.02 TgAb positivity: OR = 3.4 (1.25–9.69), *p* = 0.01 Ultrasound features: hypo-echogenicity (30.4% vs. 9.3%, *p* = 0.02) increased vascularity (35.7% vs. 5.6%, *p* = 0.001) pseudo-nodularity (16.1% vs. 0%, *p* = 0.002)
		mean age: 43.05 ± 16.72 y	
		N2 = 54 control group	
		mean age: 41.28 ± 14.78 y	
Hansen 2019 [48]	Cross-sectional study	N1 = 1127 PV	PV versus controls: similar TSH, total T4 increased total T3 (1.69 ± 0.32 vs. 1.72 ± 0.33 nmol/L; *p* = 0.01)
		mean age: 56.9 ± 12.2 y	
		N2 = 5635 controls	
		mean age: 56.9 ± 13.5 y	
Tas 2020 [53]	Cross-sectional study	N = 74 patients with psoriasis	Weak correlation between Psoriasis Quality of Life Index and thyroid diseases (r = 0.248, *p* < 0.05).
Rana 2020 [54]	Cross-sectional study	N = 290 patients with plaque psoriasis	thyroid dysfunction: 10% positive TPOAb: 13.5% similar age, gender, psoriasis severity, and duration between patients with thyroid anomalies and normal thyroid
Oktem 2020 [55]	Cross-sectional	N = 48 patients with palmoplantar pustulosis	autoimmune thyroiditis: 12%

Abbreviations: T3 = triiodothyronine; T4 = thyroxine; N = number of patients; PV = psoriasis vulgaris; vs. = versus; years = y; TPOA = antithyroperoxidase antibodies; TgAb = antithyroglobulin antibodies; TSH = Thyroid Stimulating Hormone.

**Table 3:** Original studies, aiming to analyze psoriasis in patients with thyroid diseases (2016 to 2022) [117,118,119].

Authors/ Year of Publication Reference	Type of Study	Studied Population	Results
Fallahi 2016 [117]	Prospective study	N1 = 3.069 AT	Higher prevalence of PsA in AT patients (*p* < 0.0180), nor for PsC group (*p* = 0.6237)
		mean age: 54 ± 16 y	
		N2 = 1.023 controls	
		mean age: 53 ± 15 y	
Takir 2017 [119]	Cross-sectional, controlled study	N1 = 173 with autoimmune thyroid disorders	Higher prevalence of psoriasis in N1 vs. N2 (*p* = 0.001)
		N2 = 127 with non-autoimmune conditions	
		N3 = 100 controls	
Kelada 2021 [118]	Retrospective study	N = 267 patients with thyroid eye disease	13.9% of studied population had non-thyroid autoimmunity: 3% psoriasis
		median age: 46 years	

Abbreviations: AT = autoimmune thyroiditis; N = number of patients; PsC = cutaneous psoriasis; PsA = psoriatic arthritis; y = years.

**Table 4:** Studies concerning thyroid cancer and psoriasis [121].

Authors/ Year of Publication Reference	Type of Study	Studied Population	Results
Kim 2022 [121]	Nested case-control study	N1 = 6822 subjects with thyroid cancer	TC versus controls:
		N2 = 27,288 controls	previous history of psoriasis: OR = 1.02; 95% CI 0.85–1.22

Abbreviations: N = number of patients; TC = thyroid cancer; CI = confidence interval; OR = odd ratio.

**Table 5:** Studies in oncologic patients affected by psoriasis and ATD due to anticancer drugs (from 2016 to the most recent data) [127,128,129,130,131].

Study Year Reference	Studied Population	Anti-Cancer Drug	Effects
Johnson 2016 [131]	N = 30 patients with melanoma and preexisting autoimmune disorders	ipilimumab	5/30 preexistent psoriasis 2/30 preexistent autoimmune thyroiditis (one patient died due to immune-related colitis) 27% of patients: exacerbations of autoimmune diseases
Elosua-González 2017 [130]	N = 1 patient with lung cancer	nivolumab (anti-PD1)	The patient developed de novo: palmoplantar psoriasis with nail involvement PsA autoimmune hypothyroidism
Brown 2021 [127]	N = 55 patients with melanoma and preexisting autoimmune disorders	ipilimumab and anti-PD1	flare of autoimmune diseases: 3/6 patients with psoriasis 3/19 patients with thyroiditis
Zhang 2021 [128]	N = 5560 oncologic patients (meta-analysis)	immune checkpoint inhibitors	incidental rate of thyroiditis: 0.86% 1 incidental case with psoriasis
Gonzalez-Mazón 2021 [129]	N = 102 oncologic patients (3-year, single-center experience)	immune checkpoint inhibitors	1 case de novo psoriasis 2 cases with prior psoriasis with worsening episodes 27 de novo cases with thyroiditis

Abbreviations: PD1 = programmed cell death protein; PsA = psoriatic arthritis.

## Data Availability

Not applicable.

## Abbreviations

ATD	autoimmune thyroid diseases
APS	autoimmune poly-glandular syndrome
aHR	adjusted Hazard Ratio
aOR	adjusted Odds Ratio
CRP	C reactive protein
CI	confidence interval
DPP4is	dipeptidyl peptidase-4 inhibitors
EP	erythrodermic psoriasis
EGF	Epidermal Growth Factor
fT3	free triiodothyronine
fT4	free thyroxine
GD	Graves’ disease
GPP	generalized pustular psoriasis
HLA	Human Leukocytes Antigen
HT	Hashimoto’s thyroiditis
IFN	interferon
IL	interleukin
IGF	Insulin like Growth Factor
MHC	major histocompatibility complex
OR	Odds ratio
PsA	psoriatic arthritis
PsC	cutaneous psoriasis
PP	pustular psoriasis
PV	psoriasis vulgaris
PASI	Psoriasis Area and Severity Index
SNP	Single Nucleotide Polymorphism
TNF	Tumor Necrosis Factor
TPOAb	antithyroperoxidase antibodies
TgAb	antithyroglobulin antibodies
TSH	Thyroid Stimulating Hormone
TRAb	TSH Receptor antibodies
T3	triiodothyronine
T4	thyroxine
TNFAIP3	Tumor Necrosis Factor α-induced protein 3

## References

[1] https://apps.who.int/iris/bitstream/handle/10665/204417/9789241565189_eng.pdf.psoriasis?sequence=1.

[2] Elmets C.A., Korman N.J., Prater E.F., Wong E.B., Rupani R.N., Kivelevitch D., Armstrong A.W., Connor C., Cordoro K.M., Davis D.M.R. (2021). Joint AAD-NPF Guidelines of care for the management and treatment of psoriasis with topical therapy and alternative medicine modalities for psoriasis severity measures. J. Am. Acad. Dermatol..

[3] O’Rielly D.D., Jani M., Rahman P., Elder J.T. (2019). The Genetics of Psoriasis and Psoriatic Arthritis. J. Rheumatol. Suppl..

[4] Raychaudhuri S.K., Maverakis E., Raychaudhuri S.P. (2014). Diagnosis and classification of psoriasis. Autoimmun. Rev..

[5] Ruffilli I., Ragusa F., Benvenga S., Vita R., Antonelli A., Fallahi P., Ferrari S.M. (2017). Psoriasis, Psoriatic Arthritis, and Thyroid Autoimmunity. Front. Endocrinol..

[6] Armstrong A.W., Read C. (2020). Pathophysiology, Clinical Presentation, and Treatment of Psoriasis: A Review. JAMA.

[7] Goldenstein-Schainberg C., Favarato M.H., Ranza R. (2012). Current and relevant concepts in psoriatic arthritis. Rev. Bras. Reumatol..

[8] Coates L.C., Aslam T., Al Balushi F., Burden A.D., Burden-Teh E., Caperon A.R., Cerio R., Chattopadhyay C., Chinoy H., Goodfield M.J. (2013). Comparison of three screening tools to detect psoriatic arthritis in patients with psoriasis (CONTEST study). Br. J. Dermatol..

[9] Nestle F.O., Kaplan D.H., Barker J. (2009). Psoriasis. N. Engl. J. Med..

[10] Dombrowski Y., Schauber J. (2012). Cathelicidin LL-37: A defense molecule with a potential role in psoriasis pathogenesis. Exp. Dermatol..

[11] Alwan W., Nestle F.O. (2015). Pathogenesis and treatment of psoriasis: Exploiting pathophysiological pathways for precision medicine. Clin. Exp. Rheumatol..

[12] Blauvelt A. (2008). T-helper 17 cells in psoriatic plaques and additional genetic links between IL-23 and psoriasis. J. Investig. Dermatol..

[13] Lowes M.A., Russell C.B., Martin D.A., Towne J.E., Krueger J.G. (2013). The IL-23/T17 pathogenic axis in psoriasis is amplified by keratinocyte responses. Trends Immunol..

[14] Boehncke W.H., Schön M.P. (2015). Psoriasis. Lancet.

[15] Santus P., Rizzi M., Radovanovic D., Airoldi A., Cristiano A., Conic R., Petrou S., Pigatto P.D.M., Bragazzi N., Colombo D. (2018). Psoriasis and Respiratory Comorbidities: The Added Value of Fraction of Exhaled Nitric Oxide as a New Method to Detect, Evaluate, and Monitor Psoriatic Systemic Involvement and Therapeutic Efficacy. BioMed Res. Int..

[16] Yeung H., Takeshita J., Mehta N.N., Kimmel S.E., Ogdie A., Margolis D.J., Shin D.B., Attor R., Troxel A.B., Gelfand J.M. (2013). Psoriasis severity and the prevalence of major medical comorbidity: A population-based study. JAMA Dermatol..

[17] Vashist S., Mahajan V.K., Mehta K.S., Chauhan P.S., Yadav R.S., Sharma S.B., Sharma V., Sharma A., Chowdhary B., Kumar P. (2020). Association of Psoriasis with Autoimmune Disorders: Results of a Pilot Study. Indian Dermatol. Online J..

[18] Wu J.J., Nguyen T.U., Poon K.Y., Herrinton L.J. (2012). The association of psoriasis with autoimmune diseases. J. Am. Acad. Dermatol..

[19] Malerba M., Damiani G., Radaeli A., Ragnoli B., Olivini A., Calzavara-Pinton P.G. (2015). Narrowband ultraviolet B phototherapy in psoriasis reduces proinflammatory cytokine levels and improves vitiligo and neutrophilic asthma. Br. J. Dermatol..

[20] Hsu L.N., Armstrong A.W. (2012). Psoriasis and autoimmune disorders: A review of the literature. J. Am. Acad. Dermatol..

[21] Sandru F., Carsote M., Albu S.E., Dumitrascu M.C., Valea A. (2021). Vitiligo and chronic autoimmune thyroiditis. J. Med. Life.

[22] Lee H.J., Li C.W., Hammerstad S.S., Stefan M., Tomer Y. (2015). Immunogenetics of autoimmune thyroid diseases: A comprehensive review. J. Autoimmun..

[23] Antonelli A., Ferrari S.M., Corrado A., Di Domenicantonio A., Fallahi P. (2015). Autoimmune thyroid disorders. Autoimmun. Rev..

[24] Bogusławska J., Godlewska M., Gajda E., Piekiełko-Witkowska A. (2022). Cellular and molecular basis of thyroid autoimmunity. Eur. Thyroid J..

[25] Aversa T., Corica D., Zirilli G., Pajno G.B., Salzano G., De Luca F., Wasniewska M. (2019). Phenotypic Expression of Autoimmunity in Children With Autoimmune Thyroid Disorders. Front. Endocrinol..

[26] Santos L.R., Neves C., Melo M., Soares P. (2018). Selenium and Selenoproteins in Immune Mediated Thyroid Disorders. Diagnostics.

[27] Dwivedi S.N., Kalaria T., Buch H. (2023). Thyroid autoantibodies. J. Clin. Pathol..

[28] Vastarella M., Megna M., Lupoli G.A., Napolitano M., Gallo L., Balato A., Tasso M., Costa L., Fabbrocini G., Peluso R. (2021). Is there any association between psoriasis, psoriatic arthritis and thyroid autoimmunity?. Australas. J. Dermatol..

[29] Chandran V., Cook R.J., Edwin J., Shen H., Pellett F.J., Shanmugarajah S., Rosen C.F., Gladman D.D. (2010). Soluble biomarkers differentiate patients with psoriatic arthritis from those with psoriasis without arthritis. Rheumatology.

[30] Tiniakou E., Costenbader K.H., Kriegel M.A. (2013). Sex-specific environmental influences on the development of autoimmune diseases. Clin. Immunol..

[31] Fallahi P., Ferrari S.M., Ruffilli I., Elia G., Miccoli M., Sedie A.D., Riente L., Antonelli A. (2017). Increased incidence of autoimmune thyroid disorders in patients with psoriatic arthritis: A longitudinal follow-up study. Immunol. Res..

[32] Hofman A., Brusselle G.G.O., Murad S.D., van Duijn C.M., Franco O.H., Goedegebure A., Ikram M.A., Klaver C.C., Nijsten T.E., Peeters R.P. (2015). The Rotterdam Study: 2016 objectives and design update. Eur. J. Epidemiol..

[33] Khan S.R., Bano A., Wakkee M., Korevaar T.I.M., Franco O.H., Nijsten T.E.C., Peeters R.P., Chaker L. (2017). The association of autoimmune thyroid disease (AITD) with psoriatic disease: A prospective cohort study, systematic review and meta-analysis. Eur. J. Endocrinol..

[34] Du J., Ma C., Wang R., Lin L., Gao L., Chen S., Lu X. (2021). Relationship between Different Psoriasis Types and Thyroid Dysfunction: A Retrospective Analysis. Scanning.

[35] Misiak-Galazka M., Wolska H., Galazka A., Kwiek B., Rudnicka L. (2018). General Characteristics and Comorbidities in Patients with Palmoplantar Pustulosis. Acta Dermatovenerol. Croat..

[36] Trattner H., Blüml S., Steiner I., Plut U., Radakovic S., Tanew A. (2017). Quality of life and comorbidities in palmoplantar pustulosis—A cross-sectional study on 102 patients. J. Eur. Acad. Dermatol. Venereol..

[37] Namiki K., Kamata M., Shimizu T., Chijiwa C., Uchida H., Okinaga S., Harafuji M., Nagata M., Fukaya S., Hayashi K. (2020). Thyroid dysfunction in patients with psoriasis: Higher prevalence of thyroid dysfunction in patients with generalized pustular psoriasis. J. Dermatol..

[38] Zheng J., Gao Y., Liu N., Li Y., Chen F., Yu N., Ding Y., Yi X. (2020). Higher prevalence of thyroid dysfunction in patients with erythrodermic psoriasis. J. Dermatol..

[39] Tang C., Dong Y., Lu L., Zhang N. (2021). C-reactive protein and thyroid-stimulating hormone levels as risk factors for hypothyroidism in patients with subacute thyroiditis. Endocr. Connect..

[40] Wang S.H., Wang J., Lin Y.S., Tung T.H., Chi C.C. (2019). Increased risk for incident thyroid diseases in people with psoriatic disease: A cohort study. J. Am. Acad. Dermatol..

[41] Liu J., Thatiparthi A., Martin A., Wu J.J. (2022). Association between psoriasis and thyroid dysfunction among US adults in the 2009-2014 National Health and Nutrition Examination Survey. J. Am. Acad. Dermatol..

[42] Kiguradze T., Bruins F.M., Guido N., Bhattacharya T., Rademaker A., Florek A.G., Posligua A., Amin S., Laumann A.E., West D.P. (2017). Evidence for the association of Hashimoto’s thyroiditis with psoriasis: A cross-sectional retrospective study. Int. J. Dermatol..

[43] Valduga J.A.G., Rebeiko L.B., Skare T.L. (2021). Prevalence of Hashimoto’s thyroiditis in psoriasis patients. Rev. Assoc. Med. Bras..

[44] Zhang X., Zhang S., Wu R., Li S., Su Y., Zhang P. (2022). Prevalence of autoimmune thyroid disease in patients with psoriasis: A meta-analysis. BMJ Open.

[45] Theodorakopoulou E., Yiu Z.Z., Bundy C., Chularojanamontri L., Gittins M., Jamieson L.A., Motta L., Warren R.B., Griffiths C.E. (2016). Early- and late-onset psoriasis: A cross-sectional clinical and immunocytochemical investigation. Br. J. Dermatol..

[46] Yumnam D., Kansal N.K., Kant R. (2022). Association of Psoriasis With Thyroid Disorders: A Hospital-Based, Cross-Sectional Study. Cureus.

[47] Alidrisi H.A., Al Hamdi K., Mansour A.A. (2019). Is There Any Association Between Psoriasis and Hashimoto’s Thyroiditis?. Cureus.

[48] Hansen P.R., Isaksen J.L., Jemec G.B., Ellervik C., Kanters K.J. (2019). Thyroid function in psoriasis. Br. J. Dermatol..

[49] Bergholdt H.K.M., Bathum L., Kvetny J., Rasmussen D.B.R., Moldow B., Hoeg T., Jemec G.B.E., Berner-Nielsen H., Nordestgaard B.G., Ellervik C. (2013). Study design, participation and characteristics of the Danish General Suburban Population Study. Dan. Med. J..

[50] Lai Y.C., Yew Y.W. (2016). Psoriasis and thyroid profile: Analysis of the U.S. National Health and Nutrition Examination Survey database. Indian J. Dermatol. Venereol. Leprol..

[51] Robati R.M., Toossi P., Rahmati-Roodsari M., Khalilazar S., Abolhasani E., Namazi N., Younespour S. (2013). Association of psoriasis severity with serum prolactin, thyroid hormones, and cortisol before and after treatment. Sci. World J..

[52] Vassilatou E., Papadavid E., Papastamatakis P., Alexakos D., Koumaki D., Katsimbri P., Hadjidakis D., Dimitriadis G., Rigopoulos D. (2017). No association of psoriasis with autoimmune thyroiditis. J. Eur. Acad. Dermatol. Venereol..

[53] Tas B., Kabeloglu V., Soysal A., Atakli D. (2020). Sleep Quality in Psoriasis Patients and its Relations with Possible Affecting Factors. Med. Bull. Sisli Eftal Hosp..

[54] Rana A., Mahajan V.K., Chauhan P.S., Mehta K.S., Sharma S.B., Sharma A., Sharma R. (2020). The Association of Thyroid Dysfunction with Chronic Plaque Psoriasis: A Hospital-Based Retrospective Descriptive Observational Study. Indian Dermatol. Online J..

[55] Oktem A., Uysal P.I., Akdoğan N., Tokmak A., Yalcin B. (2020). Clinical characteristics and associations of palmoplantar pustulosis: An observational study. An. Bras. Dermatol..

[56] Olazagasti J.M., Ma J.E., Wetter D.A. (2017). Clinical Features, Etiologic Factors, Associated Disorders, and Treatment of Palmoplantar Pustulosis: The Mayo Clinic Experience, 1996–2013. Mayo Clin. Proc..

[57] Braun D., Schweizer U. (2018). Thyroid Hormone Transport and Transporters. Vitam. Horm..

[58] Mancino G., Miro C., Di Cicco E., Dentice M. (2021). Thyroid hormone action in epidermal development and homeostasis and its implications in the pathophysiology of the skin. J. Endocrinol. Investig..

[59] Antonini D., Sibilio A., Dentice M., Missero C. (2013). An Intimate Relationship between Thyroid Hormone and Skin: Regulation of Gene Expression. Front. Endocrinol..

[60] Roman I.I., Constantin A.M., Marina M.E., Orasan R.I. (2016). The role of hormones in the pathogenesis of psoriasis vulgaris. Clujul Med..

[61] Utaş S., Köse K., Yazici C., Akdaş A., Keleştimur F. (2002). Antioxidant potential of propylthiouracil in patients with psoriasis. Clin. Biochem..

[62] Safer J.D. (2012). Thyroid hormone action on skin. Curr. Opin. Endocrinol. Diabetes Obes..

[63] Contreras-Jurado C., García-Serrano L., Gómez-Ferrería M., Costa C., Paramio J.M., Aranda A. (2011). The thyroid hormone receptors as modulators of skin proliferation and inflammation. J. Biol. Chem..

[64] Antonelli A., Ferrari S.M., Giuggioli D., Ferrannini E., Ferri C., Fallahi P. (2014). Chemokine (C-X-C motif) ligand (CXCL)10 in autoimmune diseases. Autoimmun. Rev..

[65] Shabgah A.G., Fattahi E., Shahneh F.Z. (2014). Interleukin-17 in human inflammatory diseases. Postepy Dermatol. Alergol..

[66] Dumitru N., Ghemigian A., Carsote M., Albu S.E., Terzea D., Valea A. (2016). Thyroid nodules after initial evaluation by primary health care practitioners: An ultrasound pictorial essay. Arch. Balk. Med. Union.

[67] Antonelli A., Fallahi P., Delle Sedie A., Ferrari S.M., Maccheroni M., Bombardieri S., Riente L., Ferrannini E. (2009). High values of Th1 (CXCL10) and Th2 (CCL2) chemokines in patients with psoriatic arthtritis. Clin. Exp. Rheumatol..

[68] Liu C., Papewalis C., Domberg J., Scherbaum W.A., Schott M. (2008). Chemokines and autoimmune thyroid diseases. Horm. Metab. Res..

[69] Júnior D.S.T. (2020). Environmental and individual factors associated with protection and predisposition to autoimmune diseases. Int J. Health Sci..

[70] Ordoñez-Cañizares M.C., Mena-Vázquez N., Redondo-Rodriguez R., Manrique-Arija S., Jimenez-Núñez F.G., Ureña-Garnica I., Fernández-Nebro A. (2022). Frequency of Polyautoimmunity in Patients With Rheumatoid Arthritis and Systemic Lupus Erythematosus. J. Clin. Rheumatol..

[71] Krishna M.T., Subramanian A., Adderley N.J., Zemedikun D.T., Gkoutos G.V., Nirantharakumar K. (2019). Allergic diseases and long-term risk of autoimmune disorders: Longitudinal cohort study and cluster analysis. Eur. Respir. J..

[72] Manvi S., Mahajan V.K., Mehta K.S., Yadav R.S., Bhushan S., Chauhan P.S. (2019). Psoriasis and Co-morbidities: Is Hyperhomocystienemia the Common Link?. J. Assoc. Physicians India.

[73] Fogel R., Comerford M., Chilukuri P., Orman E., Chalasani N., Lammert C. (2018). Extrahepatic Autoimmune Diseases are Prevalent in Autoimmune Hepatitis Patients and Their First-Degree Relatives: Survey Study. Interact. J. Med. Res..

[74] Baldini E., Odorisio T., Tuccilli C., Persechino S., Sorrenti S., Catania A., Pironi D., Carbotta G., Giacomelli L., Arcieri S. (2018). Thyroid diseases and skin autoimmunity. Rev. Endocr. Metab. Disord..

[75] Jiang Y., Mao X., Song R., Mu K., Yang Y., Zhang J.A. (2022). Psoriasis Susceptibility 1 Candidate 1 (PSORS1C1) Polymorphism is Associated with Autoimmune Thyroid Disease in a Chinese Han Population. Immunol. Investig..

[76] Hautala T., Vähäsalo P., Kuismin O., Keskitalo S., Rajamäki K., Väänänen A., Simojoki M., Säily M., Pelkonen I., Tokola H. (2021). A Family With A20 Haploinsufficiency Presenting With Novel Clinical Manifestations and Challenges for Treatment. J. Clin. Rheumatol..

[77] Solvin Å.Ø., Chawla K., Olsen L.C., Hegre S.A., Danielsen K., Jenssen M., Furberg A.S., Saunes M., Hveem K., Saetrom P. (2022). MicroRNA profiling of psoriatic skin identifies 11 miRNAs associated with disease severity. Exp. Dermatol..

[78] Krawczyk A., Miśkiewicz J., Strzelec K., Wcisło-Dziadecka D., Strzalka-Mrozik B. (2020). Apoptosis in Autoimmunological Diseases, with Particular Consideration of Molecular Aspects of Psoriasis. Med. Sci. Monit..

[79] Korkmaz S., Korkmaz H. (2017). Effect of alterations in apoptotic pathway on development of metabolic syndrome in patients with psoriasis vulgaris. Br. J. Dermatol..

[80] Laron Z., Shulman L., Hampe C., Blumenfeld O. (2023). Hypothesis: Viral infections of pregnant women may be early triggers of childhood type 1 diabetes and other autoimmune disease. J. Autoimmun..

[81] Ramos-Ruperto L., Busca-Arenzana C., Díez-Vidal A., Robles-Marhuenda Á., Díaz-Almirón M., Micán R.E., Montejano R., Valencia E., Montes M., Martin-Carbonero L. (2023). Prevalence and Temporal Trends of Autoimmune Diseases in People Living with HIV. AIDS Res. Hum. Retrovir..

[82] Yen Y.F., Chuang P.H., Jen I.A., Chen M., Lan Y.C., Liu Y.L., Lee Y., Chen Y.H., Chen Y.A. (2017). Incidence of autoimmune diseases in a nationwide HIV/AIDS patient cohort in Taiwan, 2000–2012. Ann. Rheum. Dis..

[83] Mormile I., Punziano A., Riolo C.A., Granata F., Williams M., de Paulis A., Spadaro G., Rossi F.W. (2021). Common Variable Immunodeficiency and Autoimmune Diseases: A Retrospective Study of 95 Adult Patients in a Single Tertiary Care Center. Front. Immunol..

[84] Raglianti V., Rossi G.M., Vaglio A. (2021). Idiopathic retroperitoneal fibrosis: An update for nephrologists. Nephrol. Dial. Transplant..

[85] Efe C., Torgutalp M., Henriksson I., Alalkim F., Lytvyak E., Trivedi H., Eren F., Fischer J., Chayanupatkul M., Coppo C. (2021). Extrahepatic autoimmune diseases in primary biliary cholangitis: Prevalence and significance for clinical presentation and disease outcome. J. Gastroenterol. Hepatol..

[86] Wang L., Cao Z.M., Zhang L.L., Dai X.C., Liu Z.J., Zeng Y.X., Li X.Y., Wu Q.J., Lv W.L. (2022). *Helicobacter Pylori* and Autoimmune Diseases: Involving Multiple Systems. Front. Immunol..

[87] Lin S.H., Chang Y.S., Lin T.M., Hu L.F., Hou T.Y., Hsu H.C., Shen Y.C., Kuo P.I., Chen W.S., Lin Y.C. (2021). Proton Pump Inhibitors Increase the Risk of Autoimmune Diseases: A Nationwide Cohort Study. Front. Immunol..

[88] Kridin K., Amber K., Khamaisi M., Comaneshter D., Batat E., Cohen A.D. (2018). Is there an association between dipeptidyl peptidase-4 inhibitors and autoimmune disease? A population-based study. Immunol. Res..

[89] Reichrath J., Zouboulis C.C., Vogt T., Holick M.F. (2016). Targeting the vitamin D endocrine system (VDES) for the management of inflammatory and malignant skin diseases: An historical view and outlook. Rev. Endocr. Metab. Disord..

[90] Sweta K., Freeda M.M., Lenin M. (2020). The Putative Role of Thyroid Hormones and Vitamin D on Severity and Quality of Life in Psoriasis. Int. J. Appl. Basic Med. Res..

[91] Osher E., Macaulay V.M. (2019). Therapeutic Targeting of the IGF Axis. Cells.

[92] Garmendia J.V., García A.H., De Sanctis C.V., Hajdúch M., De Sanctis J.B. (2022). Autoimmunity and Immunodeficiency in Severe SARS-CoV-2 Infection and Prolonged COVID-19. Curr. Issues Mol. Biol..

[93] Hallek M., Adorjan K., Behrends U., Ertl G., Suttorp N., Lehmann C. (2023). Long COVID Working Group of the Scientific Advisory Board within the German Medical Association. Post-COVID Syndrome. Dtsch. Arztebl. Int..

[94] Votto M., Castagnoli R., Marseglia G.L., Licari A., Brambilla I. (2023). COVID-19 and autoimmune diseases: Is there a connection?. Curr. Opin. Allergy Clin. Immunol..

[95] Qureshi N.K., Bansal S.K. (2021). Autoimmune Thyroid Disease and Psoriasis Vulgaris after COVID-19 in a Male Teenager. Case Rep. Pediatr..

[96] Ishina I.A., Zakharova M.Y., Kurbatskaia I.N., Mamedov A.E., Belogurov A.A., Gabibov A.G. (2023). MHC Class II Presentation in Autoimmunity. Cells.

[97] Sandalova T., Sala B.M., Achour A. (2022). Structural aspects of chemical modifications in the MHC-restricted immunopeptidome; Implications for immune recognition. Front. Chem..

[98] Emmungil H., İlgen U., Direskeneli R.H. (2021). Autoimmunity in psoriatic arthritis: Pathophysiological and clinical aspects. Turk. J. Med. Sci..

[99] Zakharova M.Y., Belyanina T.A., Sokolov A.V., Kiselev I.S., Mamedov A.E. (2019). The Contribution of Major Histocompatibility Complex Class II Genes to an Association with Autoimmune Diseases. Acta Nat..

[100] Wu Z., Tian E., Chen Y., Dong Z., Peng Q. (2023). Gut microbiota and its roles in the pathogenesis and therapy of endocrine system diseases. Microbiol. Res..

[101] Buhaș M.C., Gavrilaș L.I., Candrea R., Cătinean A., Mocan A., Miere D., Tătaru A. (2022). Gut Microbiota in Psoriasis. Nutrients.

[102] Thye A.Y., Bah Y.R., Law J.W., Tan L.T., He Y.W., Wong S.H., Thurairajasingam S., Chan K.G., Lee L.H., Letchumanan V. (2022). Gut-Skin Axis: Unravelling the Connection between the Gut Microbiome and Psoriasis. Biomedicines.

[103] Kierasińska M., Donskow-Łysoniewska K. (2021). Both the microbiome and the macrobiome can influence immune responsiveness in psoriasis. Cent. Eur. J. Immunol..

[104] Tizu M., Mărunțelu I., Cristea B.M., Nistor C., Ishkitiev N., Mihaylova Z., Tsikandelova R., Miteva M., Caruntu A., Sabliov C. (2022). PLGA Nanoparticles Uptake in Stem Cells from Human Exfoliated Deciduous Teeth and Oral Keratinocyte Stem Cells. J. Funct. Biomater..

[105] Fenneman A.C., Bruinstroop E., Nieuwdorp M., van der Spek A.H., Boelen A. (2023). A Comprehensive Review of Thyroid Hormone Metabolism in the Gut and Its Clinical Implications. Thyroid.

[106] Bargiel P., Szczuko M., Stachowska L., Prowans P., Czapla N., Markowska M., Petriczko J., Kledzik J., Jędrzejczyk-Kledzik A., Palma J. (2021). Microbiome Metabolites and Thyroid Dysfunction. J. Clin. Med..

[107] Silverberg N. (2022). The genetics of pediatric cutaneous autoimmunity: The sister diseases vitiligo and alopecia areata. Clin. Dermatol..

[108] Cayir A., Engin R.I., Turan M.I., Pala E. (2014). Psoriasis vulgaris and autoimmune polyendocrine syndrome type I: A case report. J. Pediatr. Endocrinol. Metab..

[109] Poojary S.A., Lodha N., Gupta N. (2015). Psoriasis in autoimmune polyendocrine syndrome type I: A possible complication or a non-endocrine minor component?. Indian J. Dermatol. Venereol. Leprol..

[110] Grossmann B., Saur S., Rall K., Pecher A.C., Hübner S., Henes J., Henes M. (2020). Prevalence of autoimmune disease in women with premature ovarian failure. Eur. J. Contracept. Reprod. Health Care.

[111] Bjørklund G., Pivin M., Hangan T., Yurkovskaya O., Pivina L. (2022). Autoimmune polyendocrine syndrome type 1: Clinical manifestations, pathogenetic features, and management approach. Autoimmun. Rev..

[112] Perniola R., Fierabracci A., Falorni A. (2021). Autoimmune Addison’s Disease as Part of the Autoimmune Polyglandular Syndrome Type 1: Historical Overview and Current Evidence. Front. Immunol..

[113] Bezzio C., Della Corte C., Vernero M., Di Luna I., Manes G., Saibeni S. (2022). Inflammatory bowel disease and immune-mediated inflammatory diseases: Looking at the less frequent associations. Therap. Adv. Gastroenterol..

[114] VanWagner L.B., Rinella M.E. (2016). Extrahepatic Manifestations of Nonalcoholic Fatty Liver Disease. Curr. Hepatol. Rep..

[115] Akiyama M., Ueno T., Kanzaki A., Kuwana M., Nagao M., Saeki H. (2016). Association of psoriasis with Hashimoto’s thyroiditis, Sjögren’s syndrome and dermatomyositis. J. Dermatol..

[116] Fichna M., Małecki P.P., Gębarski B., Gębarska H., Ruchała M. (2022). Aggregation of autoimmunity in extended families of people with autoimmune Addison disease. Intern. Med. J..

[117] Fallahi P., Ferrari S.M., Ruffilli I., Elia G., Biricotti M., Vita R., Benvenga S., Antonelli A. (2016). The association of other autoimmune diseases in patients with autoimmune thyroiditis: Review of the literature and report of a large series of patients. Autoimmun. Rev..

[118] Kelada M., Avari P., Farag S., Akishar R., Jain R., Aziz A., Feeney C., Bravis V., Meeran K., Lee V. (2021). Association of Other Autoimmune Diseases With Thyroid Eye Disease. Front. Endocrinol..

[119] Takir M., Özlü E., Köstek O., Türkoğlu Z., Mutlu H.H., Uzunçakmak T.K., Akdeniz N., Karadağ A.S. (2017). Skin findings in autoimmune and nonautoimmune thyroid disease with respect to thyroid functional status and healthy controls. Turk. J. Med. Sci..

[120] Rusiñol L., Camiña-Conforto G., Puig L. (2022). Biologic treatment of psoriasis in oncologic patients. Expert Opin. Biol. Ther..

[121] Kim S.Y., Yoo D.M., Chung J., Choi H.G. (2022). Thyroid Cancer and Psoriasis: A Nested Case-Control Study. Diagnostics.

[122] Borson-Chazot F., Borget I., Mathonnet M., Leenhardt L. (2022). SFE-AFCE-SFMN 2022 consensus on the management of thyroid nodules: Epidemiology and challenges in the management of thyroid nodules. Ann. Endocrinol..

[123] Giannoula E., Iakovou I., Giovanella L., Vrachimis A. (2022). Updated clinical management guidance during the COVID-19 pandemic: Thyroid nodules and cancer. Eur. J. Endocrinol..

[124] Apalla Z., Rapoport B., Sibaud V. (2021). Dermatologic immune-related adverse events: The toxicity spectrum and recommendations for management. Int. J. Women’s Dermatol..

[125] Calvo V., Fernández M.A., Collazo-Lorduy A., Franco F., Núñez B., Provencio M. (2021). Use of immune checkpoint inhibitors in patients with solid tumors and pre-existing autoimmune or inflammatory disease: Real-world data. Lung Cancer Manag..

[126] Danlos F.X., Voisin A.L., Dyevre V., Michot J.M., Routier E., Taillade L., Champiat S., Aspeslagh S., Haroche J., Albiges L. (2018). Safety and efficacy of anti-programmed death 1 antibodies in patients with cancer and pre-existing autoimmune or inflammatory disease. Eur. J. Cancer.

[127] Brown L.J., Weppler A., Bhave P., Allayous C., Patrinely J.R., Ott P., Sandhu S., Haydon A., Lebbe C., Johnson D.B. (2021). Combination anti-PD1 and ipilimumab therapy in patients with advanced melanoma and pre-existing autoimmune disorders. J. Immunother. Cancer.

[128] Zhang S., Zhou Z., Wang L., Li M., Zhang F., Zeng X. (2021). Rheumatic immune-related adverse events associated with immune checkpoint inhibitors compared with placebo in oncologic patients: A systemic review and meta-analysis. Ther. Adv. Chronic Dis..

[129] Gonzalez-Mazón I., Sánchez-Bilbao L., Martín-Varillas J.L., García-Castaño A., Delgado-Ruiz M., Bernat Piña I., Hernández J.L., Castañeda S., Llorca J., González-Gay M.A. (2021). Immune-related adverse events in patients with solid-organ tumours treated with immunotherapy: A 3-year study of 102 cases from a single centre. Clin. Exp. Rheumatol..

[130] Elosua-González M., Pampín-Franco A., Mazzucchelli-Esteban R., Mielgo-Rubio X., Rodriguez-Vásquez X., García-Zamora E., López-Estebaranz J.L. (2017). A case of de novo palmoplantar psoriasis with psoriatic arthritis and autoimmune hypothyroidism after receiving nivolumab therapy. Dermatol. Online J..

[131] Johnson D.B., Sullivan R.J., Ott P.A., Carlino M.S., Khushalani N.I., Ye F., Guminski A., Puzanov I., Lawrence D.P., Buchbinder E.I. (2016). Ipilimumab Therapy in Patients With Advanced Melanoma and Preexisting Autoimmune Disorders. JAMA Oncol..

[132] Furtak A., Wedrychowicz A., Starzyk J. (2020). Anti-tumour necrosis factor α therapy—Does it increase the risk of thyroid disease or protect against its development?. Pediatr. Endocrinol. Diabetes Metab..

[133] Martinez Quintero B., Yazbeck C., Sweeney L.B. (2021). Thyroiditis: Evaluation and Treatment. Am. Fam. Physician.

[134] Lee S.G., An J.H., Kim D.H., Yoon M.S., Lee H.J. (2019). A Case of Interstitial Lung Disease and Autoimmune Thyroiditis Associated with Ustekinumab. Acta Derm. Venereol..

[135] Wei Y.A., Chuang W.C., Hong C.H. (2018). Subacute thyroiditis in a patient with psoriasis treated with a tumor necrosis factor-α inhibitor. Int. J. Dermatol..

[136] Olabi B., Ayob S. (2020). Thyrotoxicosis Associated with Ustekinumab Treatment for Psoriasis. Case Rep. Dermatol. Med..

[137] Nakagawa J., Fujikawa K., Akagi M., Nakaji K., Yasui J., Hanatani Y., Hara T., Mizokami A., Kawakami A. (2021). Subacute thyroiditis in a patient with psoriatic arthritis switched from secukinumab to adalimumab: A case report and literature review. Mod. Rheumatol. Case Rep..

[138] Senlis M., Ottaviani S., Gardette A., Palazzo E., Coustet B., Dieudé P. (2017). Subacute thyroiditis in psoriatic arthritis treated by adalimumab. Jt. Bone Spine.

[139] André R., Opris A., Costantino F., Hayem G., Breban M. (2016). Cytomegalovirus subacute thyroiditis in a patient treated by infliximab for psoriatic arthritis. Jt. Bone Spine.

[140] Capo A., Amerio P. (2017). Polyglandular autoimmune syndrome type III with a prevalence of cutaneous features. Clin. Exp. Dermatol..

[141] Ben-Skowronek I., Michalczyk A., Piekarski R., Wysocka-Łukasik B., Banecka B. (2013). Type III Polyglandular Autoimmune Syndromes in children with type 1 diabetes mellitus. Ann. Agric. Environ. Med..

[142] Van Straalen J.W., de Roock S., Giancane G., Alexeeva E., Koskova E., Mesa-Del-Castillo Bermejo P., Zulian F., Civino A., Montin D., Wulffraat N.M. (2022). Prevalence of familial autoimmune diseases in juvenile idiopathic arthritis: Results from the international Pharmachild registry. Pediatr. Rheumatol. Online J..

[143] Tronconi E., Miniaci A., Pession A. (2017). The autoimmune burden in juvenile idiopathic arthritis. Ital. J. Pediatr..

[144] Sarandi E., Kruger Krasagakis S., Tsoukalas D., Rudofsky G., Tsatsakis A. (2021). A Clinical Trial for the Identification of Metabolic Biomarkers in Hashimoto’s Thyroiditis and in Psoriasis: Study Protocol. Pathophysiology.

[145] Hahn J., Cook N.R., Alexander E.K., Friedman S., Walter J., Bubes V., Kotler G., Lee I.M., Manson J.E., Costenbader K.H. (2022). Vitamin D and marine omega 3 fatty acid supplementation and incident autoimmune disease: VITAL randomized controlled trial. BMJ.

[146] Passali M., Josefsen K., Frederiksen J.L., Antvorskov J.C. (2020). Current Evidence on the Efficacy of Gluten-Free Diets in Multiple Sclerosis, Psoriasis, Type 1 Diabetes and Autoimmune Thyroid Diseases. Nutrients.

[147] Pacifico A., Conic R.R.Z., Cristaudo A., Garbarino S., Ardigò M., Morrone A., Iacovelli P., di Gregorio S., Pigatto P.D.M., Grada A. (2021). Diet-Related Phototoxic Reactions in Psoriatic Patients Undergoing Phototherapy: Results from a Multicenter Prospective Study. Nutrients.

[148] Castro P.D., Harkin G., Hussey M., Christopher B., Kiat C., Chin J.L., Trimble V., McNamara D., MacMathuna P., Egan B. (2020). Prevalence of coexisting autoimmune thyroidal diseases in coeliac disease is decreasing. United Eur. Gastroenterol. J..

[149] Conti L., Lahner E., Galli G., Esposito G., Carabotti M., Annibale B. (2018). Risk Factors Associated with the Occurrence of Autoimmune Diseases in Adult Coeliac Patients. Gastroenterol. Res. Pract..

[150] Bibbò S., Pes G.M., Usai-Satta P., Salis R., Soro S., Quarta Colosso B.M., Dore M.P. (2017). Chronic autoimmune disorders are increased in coeliac disease: A case-control study. Medicine.

[151] Wegiel M., Antosz A., Gieburowska J., Szeliga K., Hankus M., Grzybowska-Chlebowczyk U., Wiecek S., Malecka-Tendera E., Gawlik A. (2019). Autoimmunity Predisposition in Girls with Turner Syndrome. Front. Endocrinol..

[152] Kanakatti Shankar R. (2020). Immunological Profile and Autoimmunity in Turner Syndrome. Horm. Res. Paediatr..

